# Organs-on-Chips in Drug Development: Engineering Foundations, Artificial Intelligence, and Clinical Translation

**DOI:** 10.3390/bios16030155

**Published:** 2026-03-11

**Authors:** Nilanjan Roy, Luca Cucullo

**Affiliations:** 1 Department of Computer Sciences, University of Wisconsin-Madison, 1210 West Dayton Street, Madison, WI 53706, USA; nroy8@wisc.edu; 2 Department of Foundational Medical Studies, Oakland University William Beaumont School of Medicine, 586 Pioneer Dr, Rochester, MI 48309, USA

**Keywords:** organ-on-a-chip, microphysiological systems, microfluidics, biosensors, validation, artificial intelligence, multi-omics, precision medicine

## Abstract

Organ-on-a-chip (OoC) technologies, also termed microphysiological systems (MPSs), integrate microfluidics, engineered biomaterials, human-derived cells, and on-chip biosensing to model human physiology in microscale devices that deliver quantitative, time-resolved readouts. This review surveys the 2010–2025 literature, emphasizing how sensing, standardized sampling, and analytics enable clinical concordance and fit-for-purpose regulatory use. We synthesize advances in (i) materials, fabrication, and microfluidic design; (ii) organ- and disease-focused case studies; and (iii) translational benchmarks that align chip outputs with clinical pharmacokinetics, toxicology, and biomarker datasets. Across organ systems, platforms increasingly incorporate vascularization, immune components, and organoid hybrids, paired with real-time measurements of barrier integrity, metabolism, electrophysiology, and secreted biomarkers using impedance (TEER), electrochemical, and optical modalities. Representative benchmarking studies report cardiac OoCs achieving AUROC ≥ 0.85 for torsadogenic risk classification, and renal chips improving prediction of transporter-mediated clearance relative to conventional in vitro assays. We summarize validation approaches and regulatory developments relevant to new approach methodologies, including the FDA Modernization Act 2.0, and discuss how AI and multi-omics can automate signal and image analysis, harmonize cross-platform datasets, and support digital-twin workflows that couple OoC measurements to in silico models. Overall, biosensor-enabled OoCs are progressing toward quantitatively benchmarked platforms for safety pharmacology, ADME/PK–PD, and precision medicine.

## 1. Introduction

The process of drug discovery and development remains long, costly, and inefficient. The average cost of bringing a new therapeutic to market now averages around USD 2.23 billion per asset in 2024 [1], largely due to the high attrition rate in which more than 90% of candidates entering human clinical trials ultimately fail [2,3]. Despite advances in computational modeling, combinatorial chemistry, and high-throughput screening, translation of preclinical findings into successful clinical outcomes remains limited [3].

A central reason lies in the shortcomings of conventional preclinical models, both in physiological relevance and in the quality and decision-readiness of measurements they produce. Two-dimensional (2D) cell cultures are inexpensive, reproducible, and widely used, yet they lack tissue-level architecture, biochemical gradients, and biomechanical cues [4,5,6]. These oversimplifications yield drug–response data that often fail to predict organ-level toxicities or clinical efficacy, and they typically rely on sparse endpoint assays with limited capacity for continuous, non-destructive monitoring. Animal models, while more complex, exhibit significant species differences in pharmacokinetics, immunology, and disease mechanisms [7,8,9,10]. These discrepancies, combined with ethical imperatives to reduce animal use, have accelerated the search for predictive and humane alternatives [11,12]. A concise comparison of conventional 2D cell cultures, animal models, and OoC platforms is summarized in Table 1.

Recent regulatory and computational advances have reinforced this paradigm shift. The U.S. FDA Modernization Act 2.0 has explicitly opened the door for qualified non-animal methods in preclinical safety assessment [16], while emerging technical frameworks for “new approach methodologies” (NAMs) emphasize standardization, quantitative performance metrics, and fitness-for-purpose criteria [17]. In parallel, artificial intelligence (AI) and machine learning (ML) are being embedded into OoC workflows to automate image and signal analysis, fuse multi-omics datasets, and link in vitro readouts to in vivo pharmacokinetics/pharmacodynamics (PKs/PDs) and clinical endpoints [18]. Together, these developments suggest that OoC platforms can evolve from experimental curiosities into decision-relevant tools in defined contexts of use, particularly when supported by (i) transparent evidence-generation plans, (ii) quantitative benchmarking against clinical references, and (iii) measurement strategies that account for data quality considerations (e.g., calibration, drift, and reproducibility) alongside biological fidelity.

**Figure 1:**
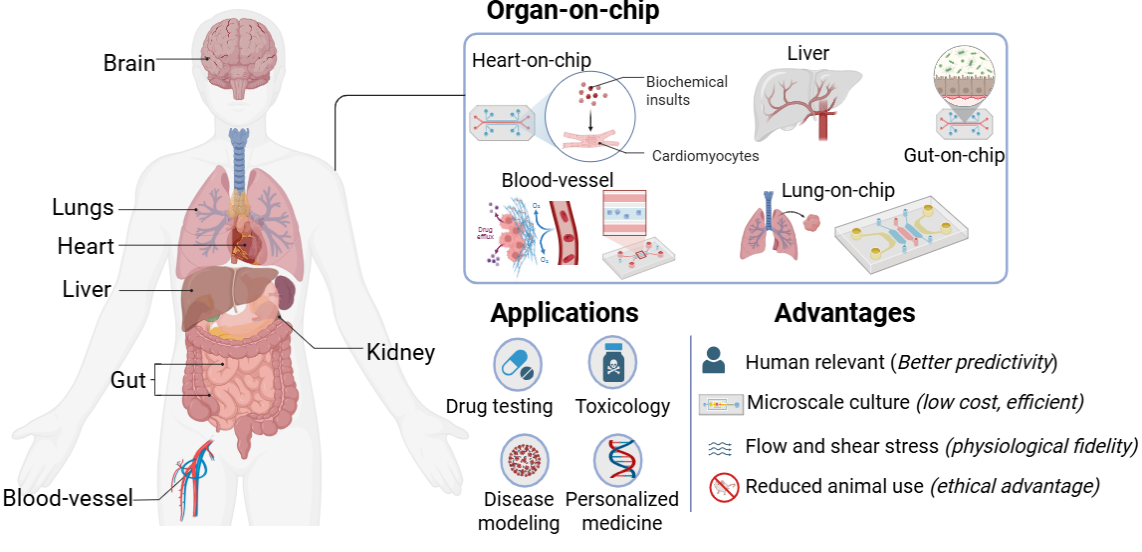
Overview of organ-on-a-chip technologies. Core fabrication strategies enable representative organ models and multi-organ platforms progressing toward multi-omics integration, clinical concordance, and regulatory adoption. *Created with BioRender.com.*

Figure 1 provides an overview of the OoC landscape, illustrating the interplay among core engineering choices (materials, microfluidic architectures, and sensing), biological inputs (primary cells, iPSCs, organoids, and immune components), and analytical and regulatory layers that determine translational impact.

Beyond addressing limitations of traditional models, OoC research has evolved rapidly over the past two decades through advances in materials, fabrication, and biological integration. Early prototypes established feasibility, whereas contemporary platforms are increasingly evaluated for scalability, reproducibility, and regulatory relevance. Table 2 outlines key milestones marking this evolution, from the advent of microfluidics to current efforts in standardization, NAM-aligned performance frameworks, and AI-enabled analytics supporting clinical concordance. Although prior reviews have examined specific aspects of OoC research, such as materials and fabrication [14,15], microfluidics [13,14], or individual organ systems [19,20,21,22,23], fewer have integrated these technological dimensions with translational and regulatory frameworks [16,17] and with AI- and multi-omics-ready measurement outputs that enable quantitative benchmarking and decision readiness [18]. This review addresses that gap by (i) evaluating advances in materials, fabrication, microfluidic control, and sensing-compatible design; (ii) surveying representative organ models across vascular, pulmonary, gastrointestinal, neurological, cardiac, renal, hepatic, cutaneous, and tumor systems, including organoid-on-chip hybrids; and (iii) highlighting how validation frameworks, quantitative benchmarks, AI-enabled analytics, and regulatory pathways are shaping adoption. By linking device-level design choices to data quality, analytically interpretable readouts, and clinical concordance, we position OoCs as potentially decision-impactful tools for safety pharmacology, ADME, and precision medicine.

## 2. Core Technologies: Engineering a Microphysiological World

The ability of organ-on-a-chip (OoC) platforms to emulate human physiology depends on four tightly coupled engineering layers: (i) materials, which define biocompatibility, sorption, gas and solute transport, and optical/electrical properties; (ii) fabrication methods, which control feature size, channel geometry, bonding, and scalability; (iii) microfluidic design, which regulates perfusion, shear stress, recirculation, and spatiotemporal gradients; and (iv) integrated biosensing and data acquisition, which convert tissue behavior into time-resolved signals that can be benchmarked, modeled, and ultimately used for decisions [13,14,15,24,25,34,35,36,37]. Iterative advances across these domains have transformed early proof-of-concept devices into increasingly standardized, manufacturable systems that can be integrated with automation, sensing, and data-analytic pipelines [14,15,34,37,38]. In this section, we highlight key material options, fabrication workflows, microfluidic strategies, and measurement considerations that underpin contemporary OoC and microphysiological system (MPS) platforms and influence translational performance.

### 2.1. Materials: The Foundation of the Chip

Material choice governs biological fidelity, reproducibility, manufacturability, and, critically for translational use, the integrity of measured signals and the stability of test compounds within the device. Key requirements include biocompatibility, suitable mechanical stiffness, controlled gas and solute permeability, optical transparency for imaging, low autofluorescence, limited sorption of hydrophobic molecules, and compatibility with microfabrication, bonding, and sensor integration [13,34,35]. No single material satisfies all constraints; instead, OoC devices increasingly use application-specific combinations of polymers, elastomers, hydrogels, and membranes [34,35,39].

- **Polydimethylsiloxane** (PDMS). PDMS remains widely used due to its elasticity, transparency, and gas permeability [13,34,36]. Its use enabled landmark devices, such as the first lung-on-a-chip, where cyclic strain simulated breathing motions [19]. However, PDMS can absorb hydrophobic drugs and small molecules, introducing variability in pharmacokinetic assays and complicating quantitative interpretation of dose–response behavior [36,39]. Surface modifications (plasma treatment, coatings, and copolymers) can reduce absorption but may compromise reproducibility or introduce additional variability over long culture durations [34,35,39].
- **Thermoplastics.** Polystyrene, polycarbonate, and cyclic olefin copolymer (COC) provide chemically inert, mass-producible alternatives [34,35,40,41]. These materials are compatible with industrial injection molding and hot embossing, allowing reproducible manufacturing at scale [34,40,41]. Their reduced drug absorption can improve PK/PD interpretability, though limited gas permeability necessitates oxygenation strategies for highly metabolic tissues [34,40,41]. Thermoplastics also facilitate standardized interfaces (ports and footprints) that support automation and consistent sensor placement across batches [40,41].
- **Hydrogels and ECM mimics.** Hydrogels, such as collagen, fibrin, gelatin methacrylate (GelMA), and polyethylene glycol (PEG), mimic extracellular matrix (ECM) stiffness and biochemical signaling [35,42,43]. They enable 3D morphogenesis, barrier formation, and immune–epithelial crosstalk. Hybrid ECM–organoid constructs have recently been combined with microfluidics to create organoid-on-chip hybrids with higher physiological fidelity and improved long-term function [42,43]. For measurement-rich workflows, hydrogel composition and remodeling kinetics can also influence diffusion and analyte transport, shaping both biological behavior and sensor readouts.
- **Hybrid and smart materials.** Multi-material composites and stimuli-responsive polymers enable dynamic tuning of stiffness or porosity [34,35,42,43]. Light- or pH-responsive substrates have been applied to mimic dynamic tissue states and trigger on-demand drug release or matrix remodeling within OoCs [34,42]. These hybrid systems aim to couple mechanical and biochemical fidelity with controlled, quantifiable perturbations that can be tracked using integrated assays.
- **Glass.** Glass substrates offer exceptional optical clarity, chemical resistance, and low drug absorption, making them attractive for imaging-intensive applications, such as blood–brain barrier and neural models [13,34,35]. Limitations include brittleness and challenges for low-cost mass manufacturing, though hybrid polymer–glass devices remain common in academic platforms [13,34]. Glass can also serve as a stable substrate for patterned electrodes and optical windows where signal stability is critical.
- **Biodegradable polymers.** Polylactic acid (PLA), polycaprolactone (PCL), and related materials are gaining attention for transient devices and remodeling–mimetic systems [34,35]. Their adoption in pharmacology remains limited by variable degradation rates, processing challenges, and the need to control degradation products that could confound biochemical measurements [34,35].
- **Paper- and textile-based substrates.** Paper-based microfluidics offers ultra-low-cost, disposable platforms with capillary-driven flow, relevant for point-of-care paradigms and low-resource settings [44]. Textile microfluidics has been piloted for wearable biosensing systems, enabling conformal contact with skin and continuous sampling of sweat or interstitial fluid [45]. While these approaches trade structural fidelity for accessibility and scalability, they provide design inspiration for disposable or wearable derivatives of OoC and sampling modules [44,45].
- **Ceramics and bioinspired composites.** Ceramics provide mechanical stability and chemical robustness, but integration into microfluidic systems is challenging. Bioinspired elastomers and conductive nanocomposites improve mechanical tunability, drug compatibility, and integration of electrical readouts in cardiac and neural OoCs [34,35,42,43].
- **Emerging 2023–2025 advances.** New bioinspired elastomers with reduced drug absorption have improved pharmacokinetic fidelity and minimized compound loss to device walls [34,36,39]. Conductive nanocomposite hydrogels enable electrical readouts in neural and cardiac tissues while preserving soft-tissue mechanics [42,43]. Multifunctional hybrid materials with tunable stiffness and sensor-compatible architectures point toward next-generation OoCs that combine mechanical fidelity with real-time quantitative readouts suitable for AI-enabled analytics [34,35,42,43].

In practice, material choices are increasingly evaluated not only for biological performance but also for data quality, including optical clarity and low autofluorescence for imaging, low sorption for PK studies, stable surface chemistry for long-term cultures, and compatibility with electrodes, probes, or imaging windows for sensor-rich experiments [17,34,35,36,42,43]. Material selection thus constrains not only tissue morphology and function but also the reliability of biochemical and omics profiles over time, which in turn influences the interpretability of downstream analytics and clinical-translational modeling [17,34,35,36,42,43].

### 2.2. Fabrication: Building Complexity at the Microscale

Fabrication governs the resolution, reproducibility, scalability, and sensor-integration readiness of OoC devices. While early platforms prioritized rapid prototyping, translation demands methods that balance architectural fidelity, throughput, and industrial manufacturability, while enabling standardized measurement interfaces. Critical requirements include micrometer-scale dimensional tolerances, robust bonding between layers, compatibility with diverse cell types, and the ability to integrate electrodes, optical access, and fluidic connectors without compromising sterility or assay stability [13,14,15,34,35,37,38,40,41].

**Figure 2:**
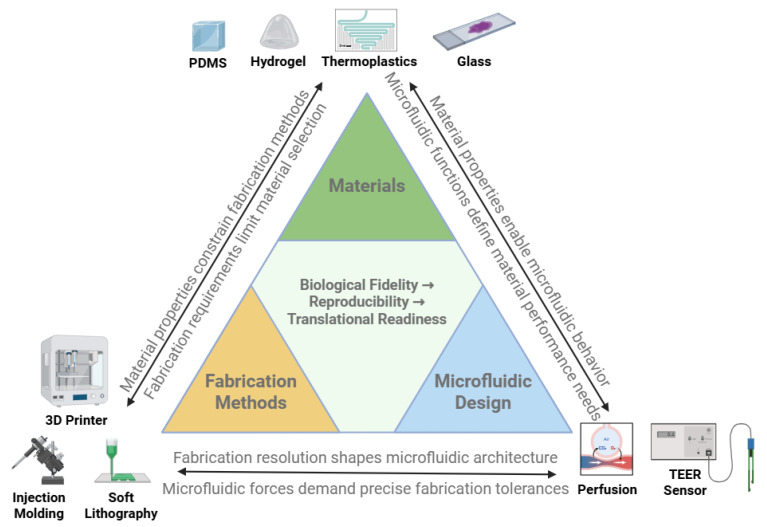
Three interdependent technological pillars, namely materials, fabrication methods, and microfluidic design, collectively determine the biological fidelity, reproducibility, and translational readiness of organ-on-a-chip systems. *Created with BioRender.com.*

- **Soft lithography.** PDMS-based soft lithography catalyzed the first wave of OoCs by offering rapid prototyping and flexible channel design [13,24,25]. Replica molding against microfabricated masters enabled controlled microchannel geometries and on-chip valves using elastomeric membranes [24,25]. However, variability between batches, limited scalability, and PDMS absorption of small molecules have restricted its translational and regulatory acceptance in some contexts [36].
- **3D printing and bioprinting.** Advances in stereolithography, two-photon polymerization, and extrusion-based printing enable fabrication of vascular networks, alveoli, and villus-like structures directly in chip formats [35,42,43]. Multi-material printing (2023–2024) can produce fine features and embed conductive or sensing-compatible elements, allowing co-fabrication of structural, soft, and conductive domains in a single workflow [35,37,42,43]. Bioprinting of cell-laden hydrogels enables spatially controlled deposition, supporting functional cardiac, neural, and vascular tissues with organoid-like architectures [35,42,43].
- **Injection molding and hot embossing.** Thermoplastic molding supports reproducible, high-throughput production at an industrial scale [34,35,40,41]. These approaches reduce inter-laboratory variability and are increasingly adopted for screening platforms [34,40,41]. Molding also enables standardized footprints, alignment guides, ports, and optical windows that improve assay reproducibility and compatibility with automated imaging and plate-based workflows [40,41].
- **MEMSs and nanofabrication.** Microelectromechanical systems (MEMSs) introduce valves, pumps, and integrated electrodes that provide automated control of flow and sensing [13,14,34,37,38]. Nanopatterning techniques replicate ECM-like topographies that influence cell alignment, migration, and barrier formation in endothelial, epithelial, and neural models [24,25,35,37]. These methods also support reproducible electrode patterning and stable interfaces for electrical readouts.
- **Design-for-manufacturing (2023–2025).** Recent work emphasizes aligning fabrication with industrial standards: dimensional repeatability, bonding stability, media compatibility, sterile packaging, and automated handling [34,37,38,40,41]. Hybrid workflows that combine academic prototyping (soft lithography and 3D printing) with industrial molding/embossing provide a practical path to innovation plus reproducibility, and they are increasingly featured in translational OoC roadmaps [34,37,38]. These fabrication requirements are summarized in Figure 2.

Ultimately, fabrication choices must satisfy both biological and operational/data constraints: devices must support long-term culture yet remain robust to automated liquid handling, high-throughput imaging, and standardized sensor readouts that can be aggregated across sites and subjected to AI/ML analysis (Section 4). Minimizing device-to-device variability in channel dimensions, surface roughness, alignment, and bonding quality reduces variability in shear stress, oxygenation, and local microenvironments, thereby stabilizing gene-expression and secretome profiles that underpin omics-based characterization and benchmarking of OoC platforms [34,35,37,38,40,41].

### 2.3. Microfluidics: Controlling the Microenvironment

Microfluidics distinguishes OoC systems from static cultures by enabling perfusion, mechanical stimulation, and systemic coupling. Proper control of microscale flows governs nutrient delivery, waste removal, biochemical gradients, and the mechanical cues that shape tissue physiology. For translational workflows, flow uniformity, shear-stress calibration, sampling consistency, and long-term stability are critical requirements for reproducibility and quantitative interpretation [13,14,34,37,38].

- **Perfusion and gradients.** Continuous flow sustains oxygenation and nutrient exchange while removing metabolic waste, supporting long-term tissue viability and barrier integrity. Controlled gradients of cytokines, oxygen, or drugs can replicate physiological or pathological microenvironments and enable mechanistic perturbation studies. Because nutrient and drug distributions shape transcriptional and metabolic states, precise microfluidic control is essential for generating reproducible multi-omics signatures comparable across devices and laboratories [13,14,34,37,38].
- Shear stress and cyclic strain. Physiological mechanical forces regulate endothelial alignment, surfactant secretion in the lung, and peristalsis-like movements in the gut [19,20,37,46]. Microfluidic actuation enables cyclic strain or pulsatile flow, allowing controlled mechanobiological dose–response mapping and improved biomimicry versus static cultures [19,20,37,46].
- Embedded sensors and real-time readouts. Integration of TEER electrodes, oxygen/pH probes, and multi-electrode arrays provides continuous readouts of barrier function, metabolism, and electrophysiology [13,23,35,37,38]. Recent advances integrate miniaturized biosensors and monitoring-compatible formats into thermoplastic devices, supporting high-throughput workflows and generating dense time-series data suitable for AI-based feature extraction and pattern recognition (Section 4.3) [34,37,38,40,41].
- Computational fluid dynamics (CFDs). CFDs guides design by predicting shear stress, diffusion, and mixing within channels, enabling optimization of geometry and flow regimes prior to fabrication [46]. Hybrid CFDs–experimental pipelines have been used to validate vascular and renal chips, reducing design iterations and improving cross-lab reproducibility [46]. These models also bridge to in silico frameworks and digital twins discussed later in multi-organ contexts.
- Closed-loop flow and systemic integration. Multi-organ systems link gut, liver, kidney, and vascular modules to model absorption, distribution, metabolism, and excretion (ADME) [14,15,22,37,38]. Recent work has demonstrated feedback-controlled perfusion regimes with embedded monitoring, supporting long-duration studies and more physiologically relevant systemic coupling [14,15,22,37,38].

**Outlook.** As OoCs mature, microfluidics is shifting from basic perfusion toward automated, feedback-controlled, multi-organ circuits. Standardization of flow rates, shear forces, media compatibility, and sampling protocols (aligned with emerging NAM guidance) will be essential for reproducibility, industrial uptake, and regulatory validation [13,14,15,16,17,18,34,37,38]. Because microfluidic design shapes the quality and structure of resulting data (imaging, electrophysiology, secretome, and omics), it sets boundary conditions for AI-enabled harmonization, feature extraction, and digital twin development described in Section 4 [16,17,18,37,38].

### 2.4. Integrated Biosensing and Measurement Validity: From Tissue Function to Decision-Ready Signals

While materials, fabrication, and microfluidics establish the biological microenvironment, integrated biosensing determines whether an OoC produces quantitative signals that are stable, comparable, and suitable for benchmarking against clinical endpoints. In translational contexts, sensor integration is not an add-on; it is a core design axis that influences what can be measured continuously, how perturbations are interpreted, and whether the platform can support standardized evidence-generation and fitness-for-purpose claims [13,16,17,18,23,35,37,38].

#### 2.4.1. What Is Typically Sensed in OoCs

Across organ systems, the most common continuous readouts include:

- **Barrier integrity and permeability** (e.g., TEER/impedance; tracer-based permeability paired with imaging) [13,23,35,37,38].
- **Metabolic state and microenvironment** (oxygen, pH, secreted factors; often paired with perfusate sampling) [13,23,35,37,38].
- **Electrophysiology and excitability** (multi-electrode array readouts in cardiac/neural contexts) [23,35,37,38].
- **Mechanical function** (contractility/strain proxies via imaging or embedded structures, depending on platform design) [19,37,46].

#### 2.4.2. Integration Constraints That Affect Signal Reliability

Sensor performance in-chip is strongly shaped by the same engineering decisions highlighted above:

- **Material interactions** (sorption, autofluorescence, surface chemistry, and swelling) that can alter analyte availability or degrade optical/electrical readouts [34,35,36,39,42,43].
- **Packaging and connectors** that introduce variability in volumes, dead space, bubbles, and contact resistances [34,37,38,40,41].
- **Biofouling and long-term culture effects,** which can shift baselines and reduce sensitivity during multi-week experiments typical of maturation or chronic exposure studies [34,35,37,38].

#### 2.4.3. Calibration, Drift, and Reproducibility as Translational Requirements

For OoCs intended for decision support, measurement practices must align with the broader push toward quantitative performance metrics and fit-for-purpose criteria in NAM frameworks [17]. At a minimum, OoC studies increasingly need to document:

- **How sensors are calibrated** (pre-run, in-run checks, or post-run validation using standards where feasible).
- **How drift/baseline shifts are handled** (e.g., reference channels, internal controls, periodic recalibration, or model-based correction).
- **How reproducibility is evaluated** (chip-to-chip, batch-to-batch, and ideally inter-site comparability when claims extend beyond a single laboratory) [17,34,37,38,40,41].

#### 2.4.4. Sampling, Signal Processing, and AI Readiness

Continuous sensing produces time-series data streams whose interpretability depends on acquisition choices:

- **Sampling strategy** (frequency, duration, and synchronization across modalities).
- **Preprocessing** (baseline correction, noise filtering, and artifact handling for bubbles/flow disruptions).
- **Feature extraction** (rates of change, recovery kinetics, oscillatory behavior, and event detection). These steps are increasingly linked to AI/ML pipelines that fuse imaging, electrophysiology, and biochemical readouts, enabling outcome prediction, phenotypic clustering, and mapping to PKs/PDs or clinical endpoints (Section 4.3) [18,37,38]. Notably, the value of AI depends on consistent measurement definitions and stable signal generation; device design choices therefore shape not only biology but also downstream analytic validity.

**Outlook.** Integrated biosensing is a key bridge between microphysiology and clinical concordance. As regulatory and industrial adoption advances, OoC platforms will be evaluated not only on whether they “look like” tissues, but on whether they produce standardizable quantitative readouts that support benchmarking, evidence generation, and clearly defined contexts of use [16,17,18]. This creates a practical convergence between chip engineering and biosensor discipline: device architectures that reduce sorption and variability, fabrication workflows that support standardized sensor placement, and microfluidics that stabilize sampling conditions collectively enable more reliable and decision-relevant OoC measurements [34,37,38,40,41].

Because the relevance of any biosensor readout is ultimately organ- and context-dependent, sensor performance in OoC platforms should be evaluated with respect to the specific physiological function being modeled rather than in isolation. Barrier organs, such as the intestine, lung, kidney, and blood–brain barrier, rely primarily on impedance- and permeability-linked measurements to capture dynamic junctional integrity, whereas excitable tissues, including the heart and nervous system, require high-temporal-resolution electrical and mechanical readouts to resolve functional electrophysiology and contractility. In contrast, metabolically active organs, such as the liver and pancreas, depend more strongly on chemical and electrochemical sensing of oxygen, nutrients, and secreted biomarkers to quantify functional state and drug response.

Table 3 synthesizes the major biosensing modalities currently integrated into OoC systems, explicitly mapping sensor type to target analytes, dominant performance-limiting factors, and translational readiness within these organ contexts. Rather than ranking technologies by nominal sensitivity, the table emphasizes practical constraints—such as signal drift, biofouling, geometry dependence, and long-term stability—that determine whether sensor outputs can support benchmarking, model validation, and decision-making workflows. This organ-aware framing highlights both the maturity of established sensing approaches (e.g., TEER in barrier models and MEAs in cardiac safety assessment) and the remaining challenges for emerging modalities as OoCs transition from exploratory platforms to translational tools. Here, translational readiness refers to the three-tier framework described in Section 5—analytical performance, biological fidelity, and clinical concordance.

## 3. Applications: Modeling Organ Function and Disease

**Figure 3:**
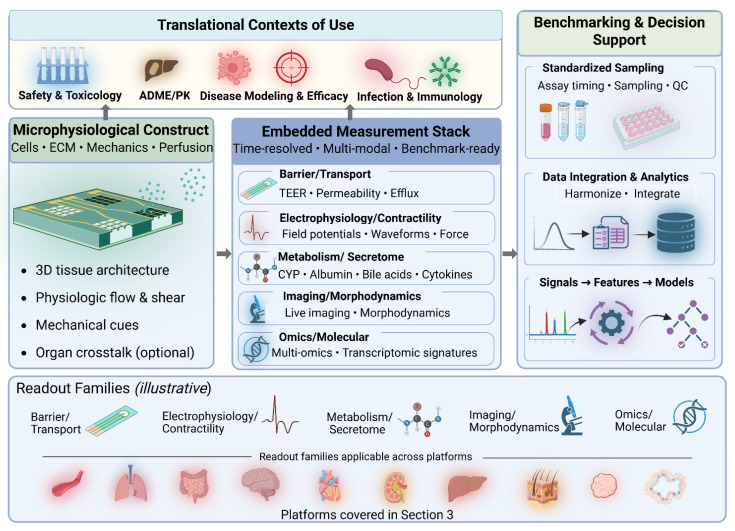
Cross-organ synthesis of organ-on-a-chip applications and readouts. Platforms are summarized as a microphysiological construct coupled to an embedded measurement stack that converts the phenotype into quantitative, time-resolved signals. Dominant readout families (barrier/transport, electrophysiology/contractility, metabolism/secretome, imaging/morphodynamics, and omics/molecular) support common translational contexts of use (safety/toxicology, ADME/PKs, disease modeling/efficacy, and infection and immunology) and, with standardized sampling and data integration, enable cross-platform benchmarking and decision support. *Created with BioRender.com.*

Organ-on-a-chip systems have been developed for multiple human organ systems. Each model leverages microengineering to reproduce essential features of physiology and pathology, enabling translational applications in drug testing, toxicology, and disease modeling [14,47,48,49,50,51,52,53,54]. For biosensors-focused translational evaluation, it is helpful to view each OoC as a microphysiological construct plus an embedded measurement stack: the biological architecture establishes the phenotype, while integrated sensing/imaging and standardized sampling convert that phenotype into quantitative, time-resolved signals suitable for benchmarking and decision support (Section 2.4). Below, we review vascular, pulmonary, gastrointestinal, neurological, cardiac, renal, hepatic, cutaneous, tumor, and organoid-on-chip platforms, highlighting mechanistic fidelity, representative case studies, and the dominant readouts used to support contexts of use. These use cases are synthesized in Figure 3 and summarized in Table 4 to highlight shared design patterns, organ-specific nuances, and dominant assay readout families [14,47,48,49,50,51,52,53,54].

### 3.1. Blood Vessel-on-a-Chip

Blood vessel chips are among the most developed OoC platforms because of the central role of the vasculature in human physiology. Endothelialized microchannels under flow reproduce barrier function, shear-stress responses, and inflammatory activation, and are now used to model vascular pathobiology across thrombosis, atherosclerosis, and microvascular disease [47,55,56,57,58].

**Dominant quantitative readouts** (typical): Endothelial barrier integrity (permeability and junctional integrity), thrombus formation kinetics under shear, inflammatory activation signatures, and trans-endothelial transport of therapeutics [47,55,56,57,58].

- **Thrombosis.** Vascular chips allow direct visualization of platelet adhesion, clot initiation, and fibrinolysis under controlled shear, including endothelium-lined “hemostasis-on-a-chip” platforms that recapitulate bleeding and thrombotic phenotypes in vitro [56,57,58].
- **Angiogenesis.** Sprouting angiogenesis has been recapitulated in collagen- or fibrin-filled channels, with interstitial flow and VEGF gradients driving lumenized vessel growth and anastomosis, enabling quantitative control of angiogenic cues [59,60].
- **Cancer metastasis.** Tumor cell intravasation and extravasation can be studied in real time within perfused microvessels, revealing how endothelial activation, matrix stiffness, and flow influence metastatic efficiency and organ tropism [47,55].
- **Drug delivery.** Vascular chips quantify trans-endothelial transport of nanoparticles, monoclonal antibodies, and small molecules and are increasingly used to benchmark permeability and vascular retention against in vivo and clinical PK datasets [47,55].
- **Recent advances (2023–2025).** Bioinspired elastomers, physiologic shear profiles, and pericyte–endothelial co-cultures now support modeling of diabetic vasculopathy, microvascular inflammation, and barrier failure, with improved alignment to patient data [47,55,57,58].

### 3.2. Lung-on-a-Chip

The lung was the first OoC successfully demonstrated, capturing the alveolar–capillary interface, with epithelial and endothelial layers separated by a porous, flexible PDMS membrane [19]. Cyclic strain simulates breathing motions, while perfusion recreates alveolar blood flow [19,20].

**Dominant quantitative readouts (typical):** Barrier integrity/leakage, cytokine dynamics, aerosol deposition/uptake, and infection kinetics under human-relevant mechanics [14,19,20,45,46].

- **Toxicology.** Lung chips replicate responses to cigarette smoke, nanoparticles, and airborne pollutants, with cytokine release and barrier breakdown matching human explant and small-airway disease data [20,45].
- **Infection.** Viral infection models (including influenza and SARS-CoV-2) have been established in advanced lung chips, enabling real-time evaluation of viral entry, replication, and antiviral therapeutics with human-relevant physiology [14,45,46].
- **Chronic lung disease.** Small-airway chips incorporating smooth muscle and immune cells model airway hyperresponsiveness, steroid sensitivity, and chronic inflammation in asthma and COPD, with readouts that correlate to patient phenotypes [20].
- **Drug delivery.** Aerosol administration modules have been integrated into lung chips, allowing quantification of droplet deposition, dissolution, and epithelial uptake under breathing-like motion and humidity [19,45].
- **Recent advances (2023–2025).** Immune-competent lung chips that incorporate circulating immune cells and patient-derived epithelium can reproduce patient-specific inflammatory signatures and therapy responses in respiratory infections and chronic airway disease [45,46].

### 3.3. Gut-on-a-Chip

Gut chips are among the most mature and widely adopted OoC systems because they reproduce villus–crypt structures, mucus production, and peristalsis-like cyclic strain under controlled flow [21,61].

**Dominant quantitative readouts (typical):** Barrier integrity (e.g., TEER/permeability surrogates), mucus and epithelial differentiation markers, microbiome-modulated functional responses, and inflammatory mediator dynamics [21,61,62,63,64,65].

- **IBD and enteropathy.** NSAID-induced enteropathy and inflammatory bowel disease (IBD) have been modeled on gut chips, with barrier disruption, cytokine release, and epithelial injury patterns reflecting patient phenotypes and clinical histopathology [21,61,62,63,64,65].
- **Drug–microbiome interactions.** Anaerobic co-culture allows integration of commensal and pathogenic bacteria, providing controlled platforms to dissect how microbiota and microbiota-derived metabolites modulate drug absorption, toxicity, and epithelial immunity [21,61,62,63,64,65].
- **Organoid integration.** Intestinal organoids seeded into microfluidic chips form villus-like morphologies with improved barrier fidelity and mucus secretion; organoid-on-chip hybrids enable long-term culture with better maintenance of stem-cell niches [42,61,62,63,64,65].
- **Multi-organ coupling.** Gut chips coupled with liver and kidney modules support modeling of first-pass metabolism and nutrient or drug transport and can be combined with PBPK modeling for translational prediction of systemic exposure [43,66].
- **Recent advances (2023–2025).** Immune-enhanced gut chips incorporating macrophages, dendritic cells, and lymphocytes enable mucosal immunity modeling, while multi-omics benchmarking aligns chip transcriptomes and epigenetic signatures with patient biopsy datasets; molecular readouts typically include tight-junction proteins (e.g., ZO-1, claudins), mucins, and inflammatory mediators that can be benchmarked against patient biopsies and multi-omics profiles [21,42,61,62,63,64,65,67].

### 3.4. Brain-on-a-Chip

Brain-on-a-chip platforms predominantly model the blood–brain barrier (BBB) and neurovascular unit, integrating iPSC-derived endothelial cells, astrocytes, pericytes, and neurons for higher physiological fidelity [22,68,69,70,71].

**Dominant quantitative readouts (typical):** The apparent permeability/efflux behavior, TEER (transepithelial/transendothelial electrical resistance) and impedance, tight-junction and transporter markers, and neural activity phenotypes (e.g., calcium imaging or MEA-style readouts) in neurovascular contexts [22,68,69,70,71].

- **Permeability.** BBB chips quantify the size- and transporter-dependent permeability of small molecules and biologics, enabling rank-order prediction of CNS penetration and comparison to in vivo datasets, including efflux transporter contributions [22,68,69].
- **Neurodegeneration.** Microengineered brain disease models combining human neurons and glia with perfused BBB interfaces capture circuit-level dysfunction and disease-relevant phenotypes, including α-synuclein pathology and barrier disruption [22].
- **Neuro-oncology.** Patient-derived glioblastoma cells interfaced with perfused BBB-like channels allow quantification of invasion, intravasation, and therapeutic penetration across an endothelial barrier under defined gradients [68,69].
- **Neuroinflammation and injury.** Hypoxia, shear stress, and inflammatory stimuli can be applied in a modular fashion to reproduce endothelial activation, tight-junction disruption, and cytokine signaling observed in stroke and neuroinflammatory conditions [22,68].
- **Recent advances (2023–2025).** Vascularized brain organoids and tissue-to-tissue BBB chips have improved nutrient delivery, maturation, and assayability, while numerical simulations and other in silico models now help optimize BBB-on-a-chip designs and flow conditions for better reproducibility [42,68,69,70,71,72]. In parallel, AI methods (such as deep learning applied to calcium imaging and multi-electrode recordings) are beginning to detect seizure-like and neuroinflammatory signatures that align with human EEG phenotypes, enabling AI-enhanced endpoint detection for CNS safety and disease modeling [18,73,74].

### 3.5. Heart-on-a-Chip

Cardiac chips use iPSC-derived cardiomyocytes seeded on patterned, elastomeric substrates, often integrated with microelectrodes to capture contractile function and electrophysiology [23,75].

**Dominant quantitative readouts (typical):** Contractile force proxies, field potential/action-potential waveform features, conduction metrics, and beat-to-beat variability [23,75].

- **Cardiotoxicity.** Heart-on-chip systems reproduce drug-induced QT prolongation, conduction block, and arrhythmias, and can outperform traditional hERG assays by integrating tissue-level repolarization and conduction heterogeneity [23,75].
- **Genetic disease.** iPSC lines from patients with inherited cardiomyopathies or channelopathies reveal contractile deficits, arrhythmic susceptibility, and differential drug responses, enabling genotype–phenotype correlation under controlled mechanical load [23,75].
- **Maturation and physiology.** Mechanical/electrical stimulation, metabolic conditioning, and matrix patterning improve tissue maturation, enabling near-adult action potentials, force generation, and calcium handling compared with conventional 2D cultures [23,75].
- **Drug discovery.** Organoid-on-chip and multi-tissue platforms integrating cardiac modules are being used for torsadogenic risk assessment and in vitro human QT prediction, benchmarked against clinical ECG data and regulatory safety pharmacology requirements [23,75]. Deep learning models trained directly on field potential or action-potential waveforms from cardiac MPS have been explored for proarrhythmic risk classification by leveraging waveform morphology and beat-to-beat variability beyond single-channel hERG assays [18,73].

### 3.6. Kidney-on-a-Chip

Kidney chips reproduce key nephron functions (filtration at the glomerulus and reabsorption/secretion in the proximal tubule) using perfused epithelial and endothelial interfaces with physiological flow [16,76,77].

**Dominant quantitative readouts (typical):** Transporter-mediated flux/clearance estimates, injury biomarkers, and epithelial function markers under physiological shear [16,76,77].

- **Nephrotoxicity.** Proximal–tubule OoCs detect dose-dependent cytotoxicity, transport-mediated injury, and drug–drug interactions, improving prediction over static cultures and supporting early flagging of nephrotoxic candidates [16,77].
- **Disease models.** Vascularized kidney organoids integrated on chips exhibit improved perfusion, transporter expression, and podocyte markers, enabling modeling of hereditary nephropathies and diabetic nephropathy under controlled hemodynamic stress [76,77].
- **Transport and clearance.** Chip-based measurements of permeability and active transport, combined with scaling and computational modeling, support in vitro–in vivo extrapolation of renal clearance and drug–metabolite handling [23,74,75].
- **Systemic coupling.** Multi-organ platforms that connect intestine, liver, and kidney modules capture metabolism–excretion interplay and enable long-term studies of ADME under controlled flow [43,66].
- **Recent advances (2023–2025).** Extended-duration perfusion with embedded sensors, tailored mechanical stimulation, and standardized readouts are improving robustness and cross-laboratory reproducibility of kidney-on-a-chip models [76,77].

### 3.7. Other Organ-on-a-Chip Models

While vascular, pulmonary, intestinal, neural, cardiac, and renal chips represent the most mature platforms, other organs have also been modeled with OoCs.

**Liver-on-a-Chip**

**Dominant quantitative readouts (typical):** Albumin/urea secretion, CYP activity panels, bile acid handling, and injury/stress–response signatures under perfusion [13,66,78].

- Supports long-term hepatocyte function (albumin, urea, and cytochrome P450 activity) under perfusion, often outperforming conventional sandwich cultures [13,66,78].
- Applied for drug-induced liver injury (DILI) prediction, mechanistic hepatotoxicity studies, and cross-species hepatotoxicity comparison, especially when coupled with non-parenchymal cell types [13,66,78].
- Coupled with gut and kidney chips, liver chips form the backbone of multi-organ ADME models for integrated PK/PD assessment [43,66].
- **2022–2024 advances.** High-throughput liver chip arrays and refined liver-on-chip designs are enabling larger compound sets, while vascularized liver organoids integrated under flow exhibit superior metabolic fidelity and disease modeling capabilities [42,66,78]. In addition to functional outputs (albumin and urea), panels of CYP isoforms, bile-acid transporters, and stress–response transcripts are increasingly used to define “hepatic fidelity” signatures that can be compared with human liver tissue and clinical DILI phenotypes [13,66,78,79].

**Skin-on-a-Chip**

**Dominant quantitative readouts (typical):** Barrier integrity surrogates, transepidermal metrics, irritation/inflammation markers, and wound closure/ECM remodeling kinetics [48].

- Recapitulates epidermal barrier function, dermal fibroblasts, and often immune competence in layered constructs, enabling barrier integrity measurements and topical exposure studies [48].
- Used for dermatology drug testing, cosmetic safety, and wound-healing studies, including re-epithelialization and scar formation assays [48].
- Recent devices incorporate perfused vasculature and immune cell layers, enabling infection and inflammation modeling and providing a more complete skin barrier-on-chip platform [47,48].

**Tumor-on-a-Chip**

**Dominant quantitative readouts (typical):** Invasion/intravasation dynamics, hypoxia/EMT signatures, cytokine/chemokine panels, and therapy–response heterogeneity from live imaging with molecular endpoints [13,42,47,49].

- Mimics the tumor microenvironment, including hypoxia, nutrient gradients, stromal support, and immune infiltration, often within ECM hydrogels under flow [13,42,47,49].
- Applied to study cancer invasion, intravasation, metastasis, and therapeutic penetration, with microfluidic vasculature enabling control over shear and vessel permeability [47,49,55].
- Immune–tumor OoCs are emerging for immunotherapy testing, capturing patient-specific immune responses and resistance mechanisms [42,47,49].
- **2023–2025 advances.** Integration of patient-derived tumor organoids with multiplexed readouts (live imaging, secretome, and omics) supports precision-oncology workflows in which therapies are screened directly on patient-matched microtumors, and the resulting high-content datasets are increasingly analyzed with deep learning to predict drug response from baseline morphology and molecular profiles, effectively turning each microtumor into an information-rich training example [18,42,47,49,66,79,80,81]. Hypoxia markers, epithelial–mesenchymal transition signatures, and immune-activation transcripts then serve as molecular endpoints that complement imaging-based invasion and killing assays in these systems [42,47,49,66].

### 3.8. Organoid-on-Chip Hybrids

Organoids derived from stem cells or patient biopsies provide self-organized, multicellular structures that recapitulate aspects of tissue physiology and disease phenotypes. However, they are limited by the absence of vascularization, immune competence, and controlled microenvironments, leading to variability and restricted scalability [42]. Integrating organoids into microfluidic OoCs addresses these challenges by combining organoid self-organization with the environmental precision of OoCs [31,42,47,70,71].

**Dominant quantitative readouts (typical):** Preservation of tissue architecture and stem-cell niches, perfusion-enabled maturation, functional barrier/transport endpoints (where relevant), and multi-omics concordance with patient samples [31,42,47,70,71].

- **Neurodevelopment and brain disease.** Cerebral organoids seeded on perfusable chips recapitulate human cortical development and, with vascularization and BBB interfaces, achieve extended viability and advanced neuronal maturation, enabling modeling of neurodevelopmental and neurodegenerative disorders [22,42,68,70,71].
- Gastrointestinal disease. Gut organoid-on-chip hybrids reproduce villus–crypt morphology, support long-term host–microbiome interactions, and provide stable platforms for modeling IBD, celiac disease, and NSAID enteropathy with multi-omics readouts [21,42,61,62,63,64,65,67].
- Metabolic and oncologic models. Pancreatic and liver organoid-on-chip systems enable modeling of diabetes and hepatic disease, while patient-derived tumor organoids incorporated into tumor chips provide precision oncology platforms where therapies can be screened directly on patient-matched microtumors [31,42,66,70,71].
- Vascularized and immune-competent constructs. iPSC-derived vascularized liver and kidney buds perfused on chips improve perfusion and transporter activity, while brain and kidney organoids in microfluidic devices show better nutrient delivery and barrier function. Immune-competent hybrids incorporating myeloid and lymphoid cells model tumor–immune interactions and viral infection, advancing OoCs toward fuller immunophysiology [22,31,42,47,66,67,70,71,77]. Endocrine (e.g., pancreatic islet) and reproductive (e.g., placenta, ovary, and testis) OoCs are also emerging and reviewed elsewhere [15,43,82].

**Table 4:** Representative organ-on-a-chip models, use cases, and assay readouts.

Organ Model	Context of Use	Key Readouts and Validation Metrics
Blood vessel	Thrombosis, angiogenesis, metastasis.	Endothelial barrier integrity; platelet adhesion and fibrin formation under defined shear; microvascular dysfunction in diabetes and inflammation.
Lung	Inhalation toxicology, viral infection, asthma/COPD.	TEER, cytokine release, barrier leakage; aerosol deposition and uptake under cyclic stretch; infection and antiviral response profiling.
Gut	IBD, NSAID enteropathy, colorectal cancer, host–microbiome.	TEER, mucus production, cytokine panels; microbiome-modulated drug response; organoid- and immune-competent gut chips benchmarked against biopsies.
Brain/BBB	BBB permeability, neurodegeneration, neuroinflammation.	Apparent permeability and efflux ratios; tight-junction markers; neural activity readouts for CNS exposure and neurotoxicity.
Heart	Cardiotoxicity, cardiomyopathies, proarrhythmia risk.	Contractile force, field potential duration, conduction velocity, and arrhythmia indices; waveform analysis for torsadogenic risk classification.
Kidney	Nephrotoxicity, diabetic nephropathy, renal clearance.	Transporter activity, transepithelial flux, and clearance estimates; injury biomarkers under physiological shear; IVIVE of renal function.
Liver	DILI, metabolic and cholestatic disease.	CYP activity, albumin and urea secretion, bile acid homeostasis; hepatotoxicity biomarkers for acute and chronic DILI prediction.
Skin	Wound healing, dermatology, topical/cosmetic safety.	Barrier TEER, transepidermal water loss, re-epithelialization and matrix remodeling; immune activation in infection and irritation models.
Tumor	Invasion, intravasation, immunotherapy response.	Invasion and intravasation metrics, tumor growth and killing assays, cytokine/chemokine panels; tumor–immune interaction profiling.
Organoid hybrids	Brain, gut, liver, kidney, tumor models; personalized testing.	Preservation of tissue architecture and stem-cell niches; vascularization; multi-omics concordance with patient samples for individualized drug response.

Representative references: blood vessel [55,56]; lung [15,19]; gut [20,61,62]; brain/BBB [21,68]; heart [22,73,75]; kidney [23,77]; liver [66,78]; skin [48]; tumor [49]; organoid hybrids [31,42,70].

Advantages of organoid-on-chip integration include improved vascularization and oxygenation, reduced variability via controlled microenvironments, support for multi-lineage co-cultures, and quantitative benchmarking through omics comparisons to patient biopsies [31,42,47,61,62,63,64,65,67,70,71]. These hybrid systems bridge the gap between self-organizing biology and engineered microenvironments, positioning OoCs as powerful tools for personalized medicine and drug discovery pipelines. Their high-content imaging and multi-omics readouts also make them natural substrates for AI-based feature extraction and multi-omics integration, as developed more fully in Section 4.3 [18]. Key design features, cell sources, and representative assay readouts for the organ-on-a-chip platforms discussed in this section are summarized above in Table 4.

## 4. Future Directions: Toward Validation, Integration, and Translation

**Figure 4:**
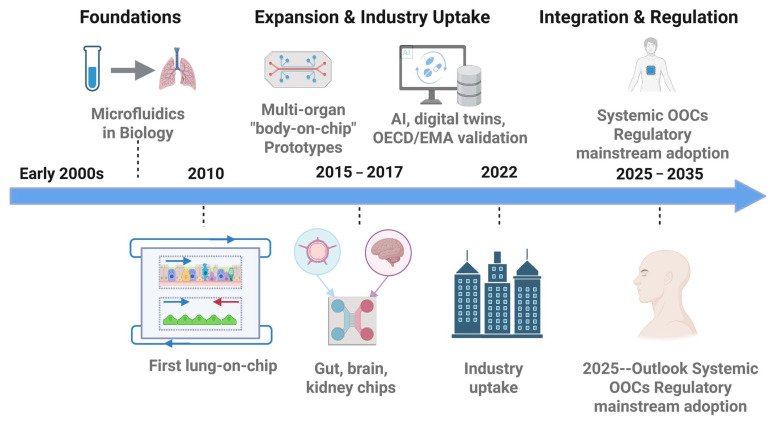
Conceptual roadmap for OoC development. Milestones from early microfluidics to automation, regulatory engagement, and multi-organ/AI-enabled platforms (2000–2035). *Created with BioRender.com.*

Organ-on-a-chip technologies have progressed from proof-of-concept devices to platforms increasingly evaluated for decision-making in discovery, safety, and early clinical translation. Recent roadmaps emphasize four intersecting trajectories: (i) deeper biological realism in individual chips, (ii) systemic integration across multiple organs and disease axes, (iii) AI- and multi-omics-enabled analytics that can extract robust, decision-relevant signals from complex datasets, and (iv) validation and regulatory frameworks that treat OoCs as quantitative tools rather than qualitative demonstrations [13,14,15,18,26,27,28,31,32,50,51,52,53,54,73,83,84,85,86,87]. For biosensors-focused translation, these trajectories are unified by a cross-cutting requirement: OoCs must be paired with an explicit measurement strategy (integrated sensing/imaging and standardized sampling that yield traceable, time-resolved outputs with known performance boundaries) (Section 2.4). A concise, fit-for-purpose checklist is summarized in Table 5, while the broader roadmap from early microfluidics to automated, multi-organ, and AI-enabled platforms is depicted in Figure 4. Together, they highlight that technological sophistication alone is insufficient; OoCs must be embedded in a data, validation, and regulatory ecosystem that allows sponsors and regulators to interpret their outputs in parallel with existing in vitro and in vivo standards [17,26,27,28,31,32,50,51,52,53,54,73,83,84,85,86,87,88,89,90,91].

### 4.1. Enhancing Biological Realism

A central challenge is to increase biological realism without sacrificing robustness and throughput. First-generation OoCs typically recapitulated one or two features of the tissue microenvironment (such as shear stress, basic barrier function, or simple cytoarchitecture), using PDMS-based devices and a limited set of cell types [13,14,15,19,24,25,34,35,36,37]. Newer designs incorporate more complex extracellular matrix (ECM) architectures, stromal and immune components, dynamic mechanical stimulation, and long-term perfusion to more closely emulate native physiology [26,27,34,35,37,38,42,43,61,70,71,85,86,87]. As realism increases, so does the need to define which phenotypes must be measured quantitatively to justify the added complexity; otherwise, more elaborate biology can simply create more variable outputs without improving decision utility [27,28,31,32,50,51,52,53,54,73,83,84,85,86,87].

From a materials perspective, development is moving away from single-material PDMS devices toward hybrid platforms that combine thermoplastics, hydrogels, and ECM-derived biomaterials to fine-tune stiffness, permeability, optical properties, and adsorption [34,35,37,38,40,41,42,43]. Multi-material 3D printing and modular assembly strategies are being used to integrate compliant membranes, porous scaffolds, and vascular-like channels into a single device while maintaining compatibility with optical imaging and plate-based workflows [26,34,35,37,38,42,43,85]. These advances support more physiologically relevant architectures (e.g., villus-like projections in gut chips, anisotropic myocardium, or aligned white-matter tracts), but they also expand the design space. In practice, fit-for-purpose design requires explicit definition of which aspects of native tissue are critical for the intended context-of-use and which can remain simplified [27,28,31,32,50,51,52,53,54,73,83,84,85,86,87].

The convergence of organoids and OoCs is another major opportunity for enhanced realism. Organoid-on-chip systems aim to retain the self-organizing, multi-lineage complexity of organoids while imposing controlled perfusion, mechanical cues, and defined gradients [21,26,27,31,42,61,62,63,64,65,67,70,71,85,86,87]. Brain and neurodevelopmental organoid-on-chip platforms now incorporate vascular surrogates, immune cells, and fluid flow to model neuroinflammation and neurotoxicity, while gut-on-chip platforms integrate microbiota and immune components to investigate barrier function and host–microbe interactions under dynamic conditions [21,26,27,42,61,62,63,64,65,67,70,71,85,86,87]. These hybrid models raise new validation questions: what constitutes acceptable variability in self-organizing systems, and which phenotypic features should be prioritized as anchors for comparison to in vivo benchmarks [26,27,85,86,87]?

Addressing these questions will increasingly require quantitative phenotyping and multi-parametric scoring rather than single-endpoint assays. This creates a direct link to AI-enabled image and signal analysis (Section 4.3), where high-content imaging, time-lapse morphodynamics, and electrophysiological readouts can be converted into composite metrics of “tissue health” or “organotypicity” that are more informative than individual markers [18,26,27,72,85,86,87,99,100,101,102,103]. For biosensors-facing adoption, an important practical implication is that biological realism should be pursued in parallel with measurement realism: stable acquisition, defined operating ranges, and standardized sampling plans that preserve interpretability as the model’s complexity increases (Section 2.4) [17,26,27,28,85,86,87,88,89,90,91,104,105,106,107].

### 4.2. From Single Organs to Systemic Models

Many early OoC applications focused on single-organ questions (such as barrier integrity, hepatotoxicity, or cardiotoxicity) that could be addressed by isolated models [13,14,15,19,20,21,22,23,31,32,50,51,52,53,54,73,83,84]. However, key translational questions in drug development and disease biology involve interactions across multiple organs and biological axes (e.g., liver–heart, gut–liver–immune, and tumor–stroma–immune), as well as special populations, such as pediatrics or pregnancy. Multi-organ and body-on-chip platforms aim to capture these interactions by linking several organ modules through recirculating media, controlled flow splitting, and physiologically inspired scaling rules for volume, surface area, and residence time [14,15,22,26,27,28,31,32,37,47,50,51,52,53,54,73,83,84,85,86,87].

This systemic integration raises several design and analysis challenges. Fluidic scaling must balance physiological relevance against practical constraints, such as total media volume, sampling requirements, and compound solubility [14,15,26,27,28,31,32,37,50,51,52,53,54,73,83,84,85,86,87]. Different organ modules may have distinct optimal media, oxygen demands, and mechanical environments, requiring compartment-specific control within a shared circuit. Interpreting outputs from multi-organ models demands clear definitions of system-level biomarkers (e.g., integrated clearance, metabolite fluxes, and distributed toxicities) rather than organ-specific readouts alone [26,27,28,31,32,50,51,52,53,54,73,83,84,85,86,87]. From a measurement perspective, this also means prioritizing system-level observables that can be collected reproducibly over time (i.e., readouts that are stable under recirculation and interpretable under changing exposure distributions) rather than only endpoint snapshots (Section 2.4) [17,26,27,28,85,86,87,88,89,90,91,104,105,106,107].

In parallel, in silico modeling of OoC devices and circuits is becoming more common, both for device design and for translational extrapolation. Computational fluid dynamics and transport models can predict shear stress, concentration gradients, and residence times across complex geometries, while multi-scale models combine chip-level pharmacokinetics with cellular pharmacodynamics to estimate exposure–response relationships [18,31,32,72,82,83,84]. New frameworks, such as DigiLoCS, explicitly couple OoC data with digital-twin representations of patients, using OoC outputs to calibrate and refine model parameters for specific scenarios [29,31,32,82,83,84]. Digital-twin-enhanced microphysiological systems have already been used to explore drug pharmacokinetics in pregnancy by linking multi-organ chips to maternal–placental–fetal PBPK models, illustrating how OoCs can be embedded in quantitative systems pharmacology workflows [31,32,82,83,84,98].

These trends point toward OoCs not as standalone replacements for animal models, but as modular components in a broader experimental–computational ecosystem. In this ecosystem, device design, flow regimens, sampling schemes, and readouts can be optimized using model-in-the-loop strategies, and chip data are interpreted through the lens of mechanistic models and digital twins that span from cell to organism [18,26,27,28,29,31,32,72,82,83,84,85,86,87,98]. AI and machine-learning (ML) methods can further support multi-organ platforms by guiding experimental design (e.g., active learning for selecting conditions), identifying informative combinations of readouts, and enabling adaptive control strategies in complex circuits (Section 4.3) [18,29,72,82,85,86,87,98,99,100,101,102,103]. For biosensors translation, a key practical point is that multi-organ “system outputs” must remain measurement-valid: stable acquisition, defined performance limits, and standardized metadata sufficient to compare circuits across sites and lots [17,26,27,28,85,86,87,88,89,90,91,104,105,106,107].

### 4.3. AI and Multi-Omics Integration

Recent innovations leverage generative machine-learning models and active-learning strategies that train on high-dimensional chip datasets to propose new compounds, experimental conditions, or design modifications. Spatial multi-omics technologies integrated with OoC platforms now enable spatially resolved transcriptomic and proteomic mapping across microfluidic architectures, helping to uncover microenvironmental heterogeneity. In addition, regulatory case studies from 2023–2024 demonstrate the FDA’s acceptance of liver- and vascular-chip data packages for toxicology and thrombosis assessments under the Modernization Act 2.0, illustrating how standardized data, transparent AI pipelines, and cross-site reproducibility can support regulatory review.

As OoCs increasingly incorporate transcriptomic, proteomic, metabolomic, and secretomic readouts, integrating these data becomes both an opportunity and a challenge. Multi-omics ML frameworks originally developed for patient cohorts (such as similarity-network fusion, multiple-kernel learning, and deep autoencoders) can be adapted to OoC datasets to identify composite signatures of toxicity, efficacy, or disease progression [30,97,108]. These approaches are well-suited to high-dimensional, heterogeneous data and can uncover latent factors that distinguish mechanisms of injury or response phenotypes, even when individual readouts are noisy [30,97,108].

In liver chips, for example, a “hepatic stress signature” might integrate CYP down-regulation, mitochondrial stress transcripts, altered bile acid transport, metabolic flux changes, and secreted cytokines. Once benchmarked against clinical or in vivo datasets, such signatures could serve as translational biomarkers for early detection of idiosyncratic hepatotoxicity or for stratifying compounds by mechanism of liver injury [18,30,31,32,50,51,52,53,54,73,83,84,97,108]. Similar strategies apply to immune–oncology, neurovascular, and gut–liver models, where multi-omics provides insight into cell–cell communication and microenvironmental remodeling [18,30,31,32,50,51,52,53,54,73,83,84,97,108]. In a biosensors context, these signatures are only as transferable as the underlying measurement pipeline: harmonized sampling windows, consistent preprocessing, and metadata that supports traceability across donors, chips, and sites (Section 2.4) [26,27,28,30,85,86,87,88,89,90,91,97,99,100,101,102,103,104,105,106,107,108,109].

**AI for imaging, morphology, and quality control.** Deep-learning models have been applied to OoC and organoid imaging for segmentation, tracking of dynamic morphological changes, and classification of treatment responses [18,99,100,101,103]. Curated organ-on-chip image datasets labeled as “acceptable” or “failed” show that convolutional neural networks can detect suboptimal tissue organization and predict downstream experimental failure from early time points [102]. Cross-platform image harmonization further enables multi-site studies by reducing batch effects across microscopes and staining protocols [99,103]. These capabilities are increasingly relevant for routine deployment: automated image-based quality control can flag chips that fall outside predefined morphological envelopes before costly downstream assays, while standardized feature representations support objective comparison across devices, laboratories, and manufacturing lots [18,26,27,28,85,86,87,99,100,101,102,103]. For decision-impact contexts, quality control should be treated as part of the measurement system, with explicitly defined acceptance thresholds and documented failure modes [17,26,27,28,85,86,87,88,89,90,91,104,105,106,107].

**Data resources, digital twins, and model-informed decisions.** Dedicated databases, such as the organs-on-a-chip database, which curate experimental metadata, device designs, cell sources, and assay results, provide a foundation for AI-driven meta-analysis and benchmarking [109]. When linked with imaging and multi-omics repositories, these resources support the construction of digital twins (computational representations of patients or subgroups calibrated using mechanistically rich OoC data) [18,26,27,28,29,30,31,32,72,82,83,84,85,86,87,97,98,99,100,101,102,103,108,109]. Recent demonstrations include pregnancy-focused digital twins that combine multi-organ chips with physiologically based pharmacokinetic models to explore drug disposition and safety in populations that are difficult to study in vivo [31,32,82,83,84,98]. More broadly, closed-loop frameworks integrating OoC experiments, ML-based surrogate models, and simulation enable efficient in silico exploration of “what-if” scenarios and experimental design before large-scale laboratory campaigns [18,29,30,31,32,72,82,83,84,97,98,108].

**Interpretability and reproducibility.** While AI-enabled workflows can increase sensitivity and throughput, they also introduce translational risk if models are not reproducible, interpretable, and robust to dataset shift across donors, sites, instruments, and device lots. In practice, OoC datasets are susceptible to confounding from batch effects (e.g., differences in imaging settings, staining protocols, cell differentiation state, or microfluidic operating conditions), which can inflate apparent performance if not explicitly controlled. For decision-impact contexts, best practices include standardized preprocessing, version-controlled pipelines (data and code), explicit reporting of training/validation splits, uncertainty quantification, and external validation on independent multi-site datasets; where feasible, interpretable representations (e.g., clinically meaningful features or mechanistic proxies) should be prioritized to support auditability and regulatory confidence [18,26,27,28,30,85,86,87,97,99,100,101,102,103,108,109]. Looking forward, AI- and multi-omics-enabled OoCs will be most impactful when embedded in transparent workflows that declare data provenance, preprocessing, model architectures, performance metrics, and limitations. For clinical and regulatory audiences, demonstrating interpretability, reproducibility across sites, and clear links between OoC-derived signatures and clinical outcomes will be as important as predictive accuracy.

### 4.4. Validation, Standards, and Regulatory Science

As OoCs move closer to regulatory and industrial decision-making, evaluation criteria are shifting from “Does the model look like an organ?” to “Does the model improve decision-making relative to current tools, for a clearly defined context of use?” [17,26,27,28,31,50,51,52,53,54,83,85,86,87,88,89,90,91]. Accordingly, validation efforts increasingly emphasize quantitative concordance with human-relevant endpoints, reproducibility across sites, and transparency of performance boundaries rather than qualitative biomimicry alone. Table 5 summarizes representative organ-specific validation metrics and target performance ranges reported in recent concordance studies and roadmap efforts. In biosensors-facing implementations, these biological performance targets should be paired with explicit measurement performance expectations (e.g., stability, sampling cadence, and comparability under standardized acquisition), because regulatory confidence depends on both the biological system and the data system that reports its state [17,26,27,28,85,86,87,88,89,90,91,104,105,106,107].

Global regulatory landscape for NAMs. Momentum toward new approach methodologies (NAMs) is increasingly global, but regulatory mechanisms and emphasis differ across jurisdictions. Agencies and consortia are converging on shared expectations: a clearly defined context of use, transparent evidence-generation plans, reproducibility (including cross-site performance where feasible), and interpretable links between assay outputs and decision-relevant endpoints. The FDA Modernization Act 2.0 has further accelerated this shift by explicitly enabling qualified non-animal methods in preclinical safety assessment, strengthening the incentive for standardized validation and reporting practices that can support regulatory confidence across regions [16,17]. Across jurisdictions, this landscape underscores the value of harmonized benchmarks, shared reference-compound panels, and transparent reporting norms to enable cross-comparability and facilitate broader acceptance of OoC-derived evidence.

Remaining challenges include integrating immune components with acceptable donor-to-donor reproducibility; scaling multi-organ systems with physiologically realistic flows, tissue ratios, and metabolic coupling; establishing consensus reference-compound panels and biomarker benchmarks; developing industrial-scale manufacturing pipelines that balance reproducibility with biological fidelity; and ensuring that AI-driven digital twins and multi-omics frameworks remain interpretable and compatible with regulatory expectations [13,14,15,18,19,20,21,22,23,26,27,28,29,30,31,32,37,47,50,51,52,53,54,61,72,73,82,83,84,85,86,87,88,89,90,91,97,98,99,100,101,102,103,104,105,106,107,108,109]. Addressing these challenges will largely determine whether OoCs achieve mainstream regulatory and industrial adoption over the next decade.

## 5. Clinical Concordance and Adoption

**Figure 5:**
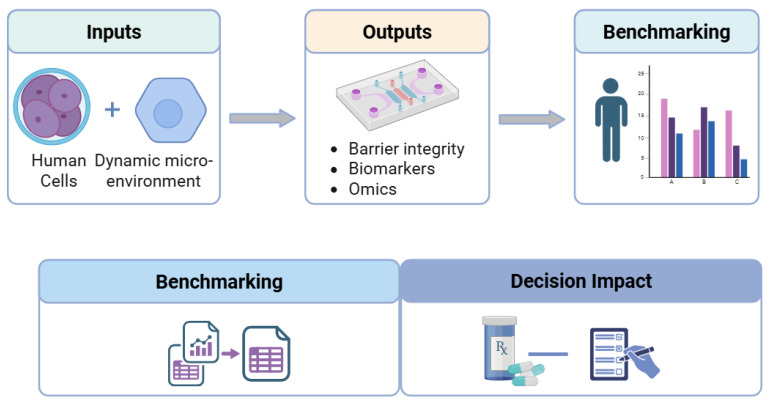
Clinical concordance workflow. Inputs (human cells and dynamic microenvironment), readouts (barrier integrity, biomarkers, electrophysiology, and multi-omics), benchmarking to patient-level datasets, and decision impact. *Created with BioRender.com*.

For organ-on-a-chip platforms to transition from academic prototypes to regulatory-accepted tools, they must demonstrate clinical concordance, defined as the ability to quantitatively reproduce patient-level outcomes and deliver decision-relevant impact. Clinical concordance goes beyond structural or molecular fidelity and requires direct benchmarking against human pharmacokinetics/pharmacodynamics (PKs/PDs), toxicology, electrophysiology, and biomarker datasets within clearly defined contexts of use. In a biosensors context, clinical concordance is only defensible when the measurement layer is characterized (e.g., acquisition stability, drift, sampling cadence, and cross-site comparability), because the “chip + sensor/imaging” system is the source of regulatory-facing evidence [27,28,29,82,88,89,90,91,98,104,105,106,107]. Recent reviews and position papers in the microphysiological systems (MPSs) and organs-on-chips field have converged on this translational framing [27,28,29,82,98]. Representative concordance outcomes are aggregated in Table 6, and the overall workflow linking chip readouts to patient data is depicted in Figure 5.

### 5.1. Defining Clinical Concordance

Validation frameworks for OoCs and related MPS typically distinguish three tiers [27,28,29,82,98]:

- **Analytical performance**, which addresses assay reproducibility, sensitivity, specificity, dynamic range, and limits of detection/quantification, including sensor/electrode performance and imaging acquisition stability (calibration, drift, and lot-to-lot comparability). This tier aligns with traditional bioanalytical validation and is essential to ensure that chip-derived measurements are technically robust across runs, operators, and sites [27,28].
- **Biological fidelity**, which compares chip-derived gene, protein, metabolic, and functional signatures to native human tissue or high-fidelity ex vivo models. Metrics include transcriptomic similarity, maintenance of tissue-specific markers, stable barrier function, and appropriate responses to positive/negative controls [27,28,82,98].
- **Clinical concordance**, which requires quantitative agreement between chip-derived outputs and patient-level outcomes, such as permeability coefficients, PK/PD parameters, QT prolongation, biomarker kinetics, and adverse event incidence [29,82,98]. This often involves in vitro–in vivo extrapolation (IVIVE), physiologically based PKs/PDs (PBPK/PDs) coupling, and comparison against historical clinical trial or real-world data [74,80,93,110].

Among these tiers, clinical concordance is the most critical for regulatory and pharmaceutical adoption because it directly addresses whether a given OoC assay improves decision-making relative to incumbent models (2D cultures, organoids, animal studies, or simple in vitro assays) [27,28,29,82,98]. Without decision-relevant alignment, even technically sophisticated platforms risk being deployed only as descriptive tools.

- **Discrepancies between chip and clinical data.** While organ-on-a-chip (OoC) systems replicate aspects of human physiology, their results may diverge from clinical outcomes for several reasons. Many early chips focus on single organs and lack systemic cross-talk; by isolating a tissue, they miss hormone, cytokine, and metabolic interactions that shape drug responses in vivo. Microfabrication materials also matter; polydimethylsiloxane (PDMS) devices absorb hydrophobic drugs, causing the actual on-chip concentration to deviate from nominal dosing [111], and drug sorption depends on hydrophobicity, flow, and medium composition. Simplified cellular composition further limits predictive power. Many chips omit vasculature and immune cells, leading to hypoxia and altered inflammatory responses [112,113]. Finally, chips built from immortalized cell lines lack the genetic and phenotypic diversity of primary human tissues [114]; without rare but clinically important subpopulations, they may mispredict treatment failures.
- **Variability among chip models.** Differences in materials, fabrication methods, and cell sourcing produce considerable variability across OoC platforms. Natural polymers such as collagen are biologically variable and depend on donor age and health, whereas synthetic polymers like PDMS are more reproducible but absorb small molecules [51,115]. Alternative materials (e.g., thermoplastics and epoxy–resin composites) offer different advantages and limitations regarding rigidity, cytotoxicity, and drug absorption [116].
- Reproducibility is also influenced by cell sourcing, such as immortalized lines, which are easy to culture but diverge from human biology, whereas primary cells better mimic physiology yet show donor-to-donor variability and limited expansion [114,117]. Autologous chips that use patient-derived tissues address genetic variability, but scaling them requires standardized protocols and culture conditions. Finally, operating conditions, such as flow rate, mechanical stress, and medium composition, differ across platforms; mismatches between static 2D and dynamic in vivo environments can lead to inconsistent responses [118]. Harmonizing materials, cell sources, and operational parameters and incorporating replicate devices to capture rare phenotypes [114] will be essential for reducing model-to-model variability.

### 5.2. Case Studies Across Organ Systems

A growing body of work now demonstrates clinical concordance across multiple organ systems, as summarized in Table 6. These examples illustrate how OoCs can reproduce human PKs, electrophysiology, toxicity, and barrier function within quantitative error bounds acceptable for decision-making.

- **Cardiac chips.** Human iPSC-derived cardiac OoCs have been benchmarked against clinical QT prolongation data and torsadogenic risk classification, with international multisite studies reporting AUROC values ≥ 0.85 and high sensitivity for borderline compounds relative to hERG-only assays [81,92]. Complementary reviews describe deep learning-enabled analytics and related organ-on-chip electrophysiology datasets that support automated waveform and image-based endpoint extraction [99,100,101,102,103]. By integrating field potential duration, contractility, and beat-to-beat variability, these systems better capture integrated cardiac responses and more accurately classify torsadogenic vs. non-torsadogenic drugs.
- **Kidney chips.** Proximal tubule-on-a-chip platforms quantify active and passive renal clearance, transporter-mediated drug–drug interactions, and nephrotoxicity, with chip-derived clearance estimates typically within 10–20% RMSE of clinical values after IVIVE scaling [74,93]. Physiologic flow and shear stress support more realistic transporter expression (e.g., OATs, OCTs, and P-gp) and improve prediction of transporter-mediated clearance compared with static cocultures [74,93].
- **Lung chips.** Lung-on-chip models reproduce key inflammatory and barrier responses in pulmonary edema, COPD, and viral infection [19,37,47]. Under cyclic stretch and air–liquid interface, they yield TEER and cytokine profiles comparable to human explants, and during COVID-19 were used to model SARS-CoV-2 infection and antiviral responses with patterns that correlated with patient data [19,37,47].
- **Liver chips.** Liver-on-a-chip platforms that maintain cytochrome P450 activity, albumin secretion, and bile acid homeostasis over weeks improve detection of both dose-dependent and idiosyncratic DILI relative to conventional 2D hepatocyte cultures [30,74,79,97]. Multi-organ MPS linking gut–liver or liver–kidney circuits further show concordance between chip-derived metabolite profiles and clinical PKs/DILI outcomes for reference compounds [74,79,105,108].
- **Brain chips and blood–brain barrier (BBB) models.** Microfluidic BBB chips comprising endothelial cells, pericytes, and astrocytes provide apparent permeability and efflux ratios for CNS-active drugs that correlate well with in vivo microdialysis or PET (R > 0.8; efflux ratios within ±10–20% of in vivo) [61,68,94,95,96]. More complex brain-on-chip systems with neuronal and glial networks recapitulate neuroinflammatory and seizure-like activity, enabling comparison of electrophysiological phenotypes to human EEG and clinical response profiles [67,73].
- **Hematologic and retinal models.** Bone marrow-on-chip platforms replicate key features of the hematopoietic niche and myelosuppression, allowing longitudinal tracking of leukocyte production and drug-induced marrow toxicity benchmarked against in vivo and clinical data [119]. Retina-on-chip devices that integrate organoid technology with microfluidic perfusion enable multi-layer retinal architectures and gene/cell therapy testing under controlled flow and oxygenation, with structural and functional metrics aligned to patient imaging and ex vivo explants [120].

### 5.3. Decision Impact and Adoption

Decision impact can be quantified using:

- Discrimination metrics, such as AUROC, sensitivity, specificity, and positive/negative predictive values (PPV/NPV).
- Calibration metrics, such as Brier scores or calibration curves comparing predicted vs. observed event rates.
- Decision-curve analysis and net benefit assessments that formally compare competing models (e.g., animal vs. OoC vs. hybrid strategies) [27,29,82,98].

In decision-curve analysis, the clinical utility of a model is evaluated across a range of risk thresholds, allowing comparison of how different testing strategies (e.g., animal models alone vs. animal plus OoCs) would change the proportion of patients correctly treated or spared unnecessary exposure [27,29,82,98]. Examples include:

- **Cardiotoxicity.** Cardiac OoCs reduce false negatives compared with hERG assays by capturing integrated electrophysiological effects (ion-channel, structural, and metabolic) [81,92,99,103]. This enables earlier deselection of unsafe compounds and refinement of dose margins.
- **Nephrotoxicity and clearance.** Kidney chips detect transporter-mediated clearance failures and nephrotoxic liabilities at preclinical stages, reducing late-stage attrition and enabling better prediction of drug–drug interactions [74,93].
- **Infectious disease and respiratory safety.** Lung chips have been used to prioritize antiviral candidates and inhaled formulations by more accurately predicting human barrier disruption and cytokine storms than traditional rodent models [19,37,47].

Pharmaceutical adoption is accelerating. Major companies have reported internal pilots or broader deployment of OoCs and MPSs for safety pharmacology, DILI prediction, and mechanistic de-risking [121,122,123,124,125]. Recent roadmaps and surveys highlight priority use cases, internal barriers to scale-up, and the need for coordinated investment in automation, data infrastructure, and AI/ML capabilities to fully exploit OoC-derived datasets [86,87,126,127,128].

Regulatory agencies and expert consortia now explicitly recognize MPS and OoCs as new approach methodologies (NAMs) for safety and efficacy assessment. Recent reviews and commentaries outline how these platforms can feed into weight-of-evidence frameworks, support mechanism-based read-across, and reduce reliance on animal models within defined contexts of use [17,91,105,127,129,130,131,132]. National and regional position papers emphasize that OoCs and related NAMs will only achieve regulatory impact if aligned with formal qualification processes, transparent reporting standards, and clearly defined performance targets for specific contexts of use [17,31,83,130,131,132].

AI- and omics-enabled OoC pipelines are increasingly positioned as key enablers of such decision impact. Integrative frameworks that couple intelligent OoC devices, high-content analytics, and multi-organ PBPKs/PDs models aim to move OoCs from exploratory tools to routine decision-support systems in both human and veterinary drug development [18,32,72,84,123,126,127,133].

### 5.4. Limitations and Barriers to Widespread Adoption

Despite rapid progress, several barriers still limit full regulatory acceptance and widespread industrial deployment of OoC platforms. These limitations span the evidence base used for benchmarking, cross-site reproducibility, study designs that demonstrate decision impact, industrial-scale manufacturing, and the integration of complex data streams (including AI-enabled analytics) into regulatory-facing workflows.

- **Incomplete reference compound libraries**. Current concordance datasets are often restricted to a limited panel of well-studied drugs, biased toward cardiotoxicity and DILI. Reference sets are sparse for rare diseases, immuno-oncology, and complex combination therapies [79,80,121,127].
- **Inter- and intra-laboratory variability.** Outcome variability persists due to differences in cell sources, media formulations, flow rates, chip geometries, and readout analysis pipelines. Recent work on standardized reporting frameworks and comparability metrics for microfluidic systems is beginning to address this, but has not yet been universally adopted [33,105,108,119,122,123,132].
- **Limited decision-impact studies.** Most publications remain descriptive, focusing on demonstrating biological relevance rather than comparative performance vs. incumbent assays. Rigorous, hypothesis-driven studies that prospectively compare OoC-informed vs. standard decision paths (e.g., using historical pipelines or simulation studies) are still relatively rare [27,29,82,93,95,96,98,119,120,127,128,130,131].
- **Industrial scaling and manufacturability.** Robust, high-throughput production of OoC devices requires scalable thermoplastic manufacturing, integrated sensing, automation, and quality systems that meet Good Laboratory Practice (GLP) expectations [80,105,108,122,134]. Many current platforms rely on PDMS or bespoke fabrication workflows that are difficult to scale or qualify.
- **Data/model integration and AI transparency.** To fully leverage clinical concordance, chip data must be integrated with PBPKs/PDs models, digital twins, multi-omics pipelines, and AI-driven analytics. Best practices for model validation, uncertainty quantification, and regulatory submission of model-informed evidence are still evolving, particularly for complex, high-dimensional OoC datasets. In addition, AI-enabled pipelines must remain reproducible and sufficiently interpretable to support confidence in high-stakes decisions [18,32,72,84,93,95,123,126,133].
- **Equity and access (cost and infrastructure constraints**). Broader translational impact will also depend on whether OoC platforms can be made affordable and operationally practical beyond highly resourced settings. Low-cost microfluidic strategies and simplified platforms (e.g., paper- or textile-inspired approaches) highlight possible pathways toward wider accessibility and neglected-disease relevance without requiring fully automated, high-instrumentation workflows [44,45].

Clinical concordance thus represents the highest tier of OoC validation, defined by quantitative agreement between chip-derived readouts and patient-level outcomes. Demonstrating this alignment establishes confidence that OoCs can replace or supplement animal and static in vitro assays within defined regulatory contexts of use. As summarized in Table 6, concordance has now been shown across heart, kidney, lung, liver, brain, and emerging hematologic and retinal systems [30,74,79,80,81,89,90,91,92,94,97,99,100,101,102,103,106,107,108,109,110,119,120,135]. Continued harmonization of validation standards, expansion of reference drug libraries, cross-laboratory benchmarking initiatives, and integration with AI-enabled, multi-omics-driven modeling frameworks will be essential to sustain this translational trajectory and enable regulatory confidence in OoC-based decision frameworks [29,30,33,93,95,96,98,119,120,121,122,123,124,125,126,127,128,129,130,131,132,133,134].

## 6. Conclusions

Organ-on-a-chip technologies have progressed from proof-of-concept microfluidic devices to translational platforms with integrated sensing, long-term perfusion, and human-derived tissues. These advances enable quantitative, time-resolved readouts of barrier integrity, metabolism, electrophysiology, and secreted biomarkers across diverse organs, and have yielded growing evidence of clinical concordance—cardiac chips achieve AUROC ≥ 0.85 for torsadogenic risk; kidney, lung, liver, and brain/blood–brain barrier models reproduce clearance, barrier, cytokine, and permeability responses, while multi-organ systems coupled with PBPKs/PDs modelling link microfluidic outputs to whole-body exposure–response relationships.

As data volumes increase, AI and multi-omics methods are essential for high-content analysis but must be embedded in transparent measurement chains to ensure reproducibility, interpretability, and regulatory confidence. Broad adoption of OoC technologies will therefore hinge on standardized engineering and manufacturing, fit-for-purpose validation frameworks, interpretable AI and omics pipelines, physiologically realistic multi-organ and immune-competent systems, and supportive data and ecosystem infrastructure. When aligned with these principles, OoCs can reduce reliance on animal models, de-risk drug development, and enable more personalized and mechanism-informed therapy.

Looking ahead, broad adoption of OoC technologies will depend less on incremental technical novelty and more on closing several translational gaps. These include:

- Standardized engineering and manufacturing—harmonized materials, fabrication workflows, and microfluidic parameters that support industrial-scale, quality-controlled production.
- Robust, transparent validation frameworks—fit-for-purpose criteria spanning analytical performance, biological fidelity, and clinical concordance, supported by structured reporting standards and shared reference compound panels.
- Integrated AI and multi-omics that are interpretable and auditable—algorithms and data pipelines that can be explained, benchmarked, versioned, and stress-tested for bias and robustness in regulatory settings.
- Physiologically realistic multi-organ and immune-competent systems—platforms that maintain stable, human-relevant interactions across tissues, immune compartments, and microbiome where appropriate.
- Data and ecosystem infrastructure—curated databases, open benchmarking studies, and cross-site collaborations that enable reproducibility, meta-analysis, and continuous refinement of models and digital twins.

Actionable recommendations for the next 2–3 years include:

- Prioritize regulatory engagement and invest in cardiac, liver, and kidney chips, which currently show the strongest clinical concordance and are most likely to gain regulatory acceptance in the near term.
- Develop and validate high-fidelity TEER and multi-sensor platforms for barrier tissues (gut, lung, and brain) to provide standardized benchmarks for immune-competent and multi-organ chips.
- Integrate AI-driven generative models and active learning to streamline experimental design and identify informative readout combinations, while ensuring pipelines are transparent, version-controlled, and interpretable.
- Expand collaborations around spatial omics integration and reference-compound panels to harmonize data across platforms and sites.

By explicitly linking engineering innovation to quantitative clinical concordance and AI-enabled analysis, OoCs are positioned to reduce reliance on animal testing, de-risk development pipelines, and enable more personalized, mechanism-informed therapy selection. Organs-on-chips now sit at the intersection of engineering, computational modeling, and clinical science; their ultimate success will be measured not by how closely they mimic native tissues in isolation, but by how reliably they improve human outcomes and reduce development risk across the therapeutic lifecycle.

## Figures and Tables

**Table 1:** Comparison of preclinical research models.

Feature	2D Cell Culture [4,5,6]	Animal Models [7,8,11,12]	Organ-on-a-Chip (OoC) [13,14,15]
Physiological relevance	Low; flat, static monolayers	High systemic complexity but non-human physiology	Medium–high; 3D microenvironments with flow and mechanical cues
Human-specific data	Yes, when human cells are used	No; inter-species differences	Yes; primary cells, iPSCs or organoids from human donors
Throughput and cost	High throughput; low cost	Low throughput; very high cost	Medium throughput; moderate cost
Ethical considerations	Minimal	Significant ethical and welfare concerns	Minimal; potential to reduce animal use
Real-time monitoring	Limited; mostly endpoint assays	Limited; many assays invasive or terminal	High; compatible with integrated sensors and live imaging
Control of variables	High control over media and stimuli	Low; complex whole-organism variability	Very high; precise control of flow, gradients, and co-cultures
Translational concordance	Often poor correlation with clinical outcomes	Variable; limited for many human-specific diseases and PK/PD	Growing evidence of quantitative concordance with human PK/PD and clinical biomarkers in defined contexts of use

**Table 2:** Representative milestones in the development of organ-on-a-chip (OoC) technology.

Year	Milestone	Reference
Early 2000s	Emergence of microfluidics for biology and organ-mimetic microdevices	[13,24,25]
2010	First lung-on-a-chip (PDMS-based, cyclic strain) demonstrating organ-level breathing functions	[19]
2012–2014	Expansion to gut, brain, and kidney chips, illustrating versatility across multiple organ systems	[20,21,23]
2015–2017	Development of multi-organ and interconnected OoC platforms enabling systemic PK/PD modeling	[14,15,26,27]
2018–2020	Growing industrial uptake, early commercialization, and international roadmaps for OoC and MPS adoption	[27,28,29,30]
2022	FDA Modernization Act 2.0 formally recognizes validated non-animal models, including OoCs, in drug development	[16]
2023–2025	AI-enabled digital twins, NAM-focused technical frameworks, and standardization efforts supporting quantitative benchmarking and regulatory acceptance	[17,18,31,32,33]

**Table 3:** Organ-specific integration of biosensors in organ-on-chip platforms: analytes, performance limits, and translational readiness.

Organ System	Sensor Modality	Primary Analyte(s)/Functional Endpoint(s)	Key Performance Limits in OoC Context	Translational Readiness
Barrier organs (gut, lung, kidney, BBB)	TEER/electrical impedance	Barrier integrity; tight junction dynamics; permeability surrogates	Absolute TEER values are highly geometry- and electrode-dependent; sensitive to flow, temperature, and bubble formation; long-term drift due to electrode polarization and fouling	High (within-platform): Widely adopted for dynamic benchmarking; limited cross-platform comparability
	Spatial or multi-frequency impedance (EIS)	Barrier heterogeneity; capacitive vs resistive components	Increased modeling and instrumentation complexity; interpretation sensitive to electrode placement and analysis assumptions	Moderate: Valuable mechanistic insight; less standardized
	Electrochemical pH/ion sensors	Local microenvironment changes affecting barrier function	Reference electrode stability; flow-induced noise; drift under long-term perfusion	Low–moderate
Cardiac	Microelectrode arrays (MEAs)	Field potentials; beat rate; conduction velocity; arrhythmogenic events	Signal amplitude depends on cell–electrode coupling; noise susceptibility; requires standardized analysis pipelines	High: Increasingly integrated into safety pharmacology workflows
	Mechanical/contractility sensors (cantilevers, strain gauges, and flexible diaphragms)	Contractile force; beat regularity; excitation–contraction coupling	Calibration drift; material fatigue; batch-to-batch variability; mechanical mismatch with tissue	Moderate–high: Strong for cardiac-specific endpoints
	Optical Ca^2+^ indicators	Excitation–contraction dynamics	Photobleaching; phototoxicity; reliance on dyes or reporters	Moderate
Neural/brain	MEAs (planar or 3D)	Network activity; firing rate; synchrony; conduction	Long-term signal stability; electrode encapsulation by cells; complex data interpretation	Moderate: Widely used in research; translational benchmarks emerging
	Neurochemical electrochemical sensors	Neurotransmitters; metabolic markers	Biofouling; selectivity in complex media; limited multiplexing	Low–moderate
Liver/metabolic organs	Electrochemical metabolite sensors (O_2_, glucose, and lactate)	Metabolic activity; mitochondrial function; drug-induced stress	Enzyme degradation; recalibration needs; sensitivity to medium composition	Moderate: Robust for core analytes with proper QC
	Optical oxygen sensors	Oxygen gradients; consumption rates	Photobleaching (intensity-based); sensor aging; spatial averaging effects	High: One of the most mature chemical sensing approaches in OoCs
	Affinity biosensors (immuno-/aptamer-based)	Cytokines; secreted injury markers	Non-specific binding; regeneration limits; matrix effects	Emerging
Cross-organ/multi-organ systems	Flow and pressure sensors	Shear stress; perfusion stability	Calibration across viscosities; control–loop instability	High: Essential enablers for credible long-term studies
	Hybrid on-chip sensing and off-chip analytics	Broad metabolite and proteomic panels	Loss of temporal resolution; dead volume; sampling perturbations	Moderate–high

**Table 5:** Representative validation metrics for OoCs.

Organ Model *	Context of Use	Key Validation Metrics
Heart	Torsadogenic risk prediction	AUROC ≥ 0.85 reported in multisite benchmarks; sensitivity ≥ 90% vs. clinical QT datasets
Kidney	Transporter-mediated clearance; nephrotoxicity	Clearance R^2^ > 0.8; RMSE ~ 10–20% vs. clinical clearance
Brain/BBB	BBB permeability and efflux	Permeability rank correlation R > 0.8; efflux ratio within ±10–20% of in vivo
Lung	Inhalation toxicology; cytokine release; infection	Stable TEER (>95% of baseline); cytokine changes within ex vivo ranges
Liver	Drug-induced liver injury (DILI) prediction	CYP activity within ±20% of in vivo; albumin and urea in physiological range
Multi-organ (ADME/PBPK)	ADME/PKs–PDs; digital-twin integration	Plasma C–t R^2^ > 0.85; prediction error < 25% for C_max and AUC

* Targets in Table 5 are supported by the case studies in Section 5; representative references: heart [81,92], kidney [74,93], BBB [61,68,94,95,96], lung [19,37,47], liver [30,74,79,97], multi-organ ADME/PKs [74,98].

**Table 6:** Clinical concordance of organ-on-a-chip models across major organ systems.

Organ	Clinical Endpoint/Comparator	Key Concordance Metrics
Heart-on-a-chip	Torsadogenic risk, QT prolongation; hERG assay and clinical QT/ECG.	Representative studies report AUROC typically ≥0.85 with sensitivity > 90% for torsadogenic drugs; improved detection of borderline-risk compounds vs. hERG alone.
Kidney-on-a-chip	Renal clearance, transporter-mediated DDI, nephrotoxicity; clinical PK and DDI; 2D cocultures.	Total and transporter-mediated clearance within ~10–20% RMSE of clinical values after IVIVE; earlier flagging of nephrotoxicity than 2D cocultures.
Lung-on-a-chip	Barrier integrity, cytokine release, viral infection; human explants, BAL/biopsy, rodent data.	TEER and cytokine responses within roughly two-fold of ex vivo human data; correct ranking of inflammatory stimuli and antiviral candidates under physiological stretch and ALI.
Liver-on-a-chip	DILI risk, metabolic function (CYP, bile acids); clinical DILI cases and human PK; 2D hepatocytes.	Maintains hepatic function for ≥3–4 weeks; improved sensitivity/specificity for clinical DILI and more accurate classification of high-risk compounds than 2D hepatocytes.
Brain/ BBB-on-a-chip	BBB permeability and efflux; neurophysiology; microdialysis, PET, and EEG data.	Permeability rank correlations R > 0.8 and efflux ratios within ±10–20% of in vivo; seizure-like and neuroinflammatory phenotypes consistent with clinical EEG signatures.
Multi-organ/systemic MPS	Plasma concentration–time curves, multi-organ toxicity; clinical PKs/PDs and reference drugs.	Cmax and AUC prediction errors generally <25% vs. clinical PKs; correct ordering of systemic toxicities and exposure–response profiles in representative compound panels.

Abbreviations: ALI, air–liquid interface; AUC, area under the concentration–time curve; AUROC, area under the receiver operating characteristic curve; BAL, bronchoalveolar lavage; BBB, blood–brain barrier; Cmax, maximum plasma concentration; CYP, cytochrome P450; DDI, drug–drug interaction; DILI, drug-induced liver injury; ECG, electrocardiogram; EEG, electroencephalography; hERG, human Ether-à-go-go–related gene; IVIVE, in vitro–in vivo extrapolation; MPS, microphysiological system; PET, positron-emission tomography; PKs, pharmacokinetics; PKs/PDs, pharmacokinetics/pharmacodynamics; QT, QT interval; RMSE, root-mean-square error; TEER, transepithelial electrical resistance. Representative references supporting the benchmarks in Table 6 are provided in Section 5; see organ-specific concordance case studies and validation examples cited therein.

## Data Availability

Not applicable.

## Abbreviations

The following abbreviations are used in this manuscript:

**Abbreviation**	**Definition**
ADME	Absorption–distribution–metabolism–excretion
ADME–Tox	Absorption–distribution–metabolism–excretion–toxicity
AI	Artificial intelligence
ALI	Air–liquid interface
AUC	Area under the concentration–time curve
AUROC	Area under the receiver operating characteristic curve
BAL	Bronchoalveolar lavage
BBB	Blood–brain barrier
CFDs	Computational fluid dynamics
Cmax	Maximum plasma concentration
CNS	Central nervous system
COC	Cyclic olefin copolymer
COPD	Chronic obstructive pulmonary disease
CYP	Cytochrome P450
DDI	Drug–drug interaction
DILI	Drug-induced liver injury
DigiLoCS	Digital Liver-on-Chip Simulator
ECG	Electrocardiogram
ECM	Extracellular matrix
EEG	Electroencephalography
FDA	U.S. Food and Drug Administration
GLP	Good Laboratory Practice
GIVReSts	Good In Vitro Reporting Standards
hERG	human Ether-à-go-go–related gene
IBD	Inflammatory bowel disease
iPSC	Induced pluripotent stem cell
IVIVE	In vitro–in vivo extrapolation
MDAR	Materials Design Analysis Reporting
MEA	Multi-electrode array
MEMSs	Microelectromechanical systems
MIAME	Minimum Information About a Microarray Experiment
ML	Machine learning
MPSs	Microphysiological system(s)
NAM	New approach methodology
NSAID	Nonsteroidal anti-inflammatory drug
OAT	Organic anion transporter
OCT	Organic cation transporter
OoC	Organ-on-a-Chip
PBPK	Physiologically based pharmacokinetic modeling
PBPK/PD	Physiologically based pharmacokinetic/pharmacodynamic modeling
PCL	Polycaprolactone
PDs	Pharmacodynamics
PDMS	Polydimethylsiloxane
PEG	Polyethylene glycol
P-gp	P-glycoprotein
PET	Positron emission tomography
PK	Pharmacokinetics
PKs/PDs	Pharmacokinetics/pharmacodynamics
PLA	Polylactic acid
QC	Quality control
QT	QT interval
RMSE	Root-mean-square error
TEER	Transepithelial electrical resistance
VEGF	Vascular endothelial growth factor
